# Prevalence and prognostic value of ventricular conduction delay in heart failure with preserved ejection fraction

**DOI:** 10.1016/j.ijcha.2025.101622

**Published:** 2025-01-24

**Authors:** Anouk Achten, Jerremy Weerts, Johan van Koll, Mohammed Ghossein, Sanne G.J. Mourmans, Arantxa Barandiarán Aizpurua, Antonius M.W. van Stipdonk, Kevin Vernooy, Frits W. Prinzen, Hans-Peter Brunner-La Rocca, Christian Knackstedt, Vanessa P.M. van Empel

**Affiliations:** a Department of Cardiology, Cardiovascular Research Institute Maastricht (CARIM), Maastricht University Medical Centre (MUMC+), Maastricht, the Netherlands; b Department of Physiology, Cardiovascular Research Institute Maastricht (CARIM), Maastricht University, Maastricht, the Netherlands

**Keywords:** Heart failure with preserved ejection fraction, Conduction delay, Electrocardiography, Prognosis

## Abstract

**Background:**

The pathophysiology of heart failure (HF) with preserved ejection fraction (HFpEF) is heterogeneous and incompletely understood. This study evaluated the presence of a ventricular conduction delay (VCD) phenotype in HFpEF through QRS duration and vectorcardiographic QRS area, and their relation to adverse outcomes.

**Methods:**

This study included consecutive ambulatory HFpEF patients. Baseline QRS duration was obtained from an electrocardiogram (ECG). QRS area was derived from vectorcardiographic analyses of the ECG. QRS duration and area were assessed and analysed as categorical (<100 ms, 100–119 ms, ≥120 ms; ≤ 43.1 µVs, >43.1 µVs) and continuous variables to determine the relation to the composite outcome of HF hospitalisation and all-cause mortality.

**Results:**

349 HFpEF patients were included of whom 70 % had a QRS duration < 100 ms compared to 21 % with QRS duration 100–119 ms and 9 % with QRS duration ≥120 ms. 87 (25 %) patients had QRS area >43.1 µVs. Only 4 % had a QRS area ≥69µVs, indicating delayed lateral wall activation. After a median of 3 years follow-up, 30 % of the patients had an adverse outcome. Longer QRS duration but not larger QRS area was associated with more adverse outcomes on both categorical and continuous scales (HR per 5 ms increase = 1.06, P = 0.033). This prognostic association was mainly present in males.

**Conclusion:**

HFpEF patients have a low prevalence of a VCD phenotype(9 % QRS duration ≥120 ms;4 % a QRS area ≥69 µVs). However, QRS duration >100 ms was present in 30 % and was an independent predictor for adverse outcomes. Future efforts are needed to understand the mechanisms underlying the association of QRS duration and adverse outcomes, and to determine its clinical implications.

## 1 Introduction

Heart failure with preserved ejection fraction (HFpEF) affects at least one-half of patients with chronic heart failure (HF) [1], [2], [3]. Despite the high prevalence of HFpEF, the pathophysiology remains controversial and effective therapies are scarce [4]. Identifying characteristics to subcategorize HFpEF is a promising approach to determine the underlying pathophysiological mechanisms and to improve treatment. A group of pathophysiological similar patients with HFpEF may respond in a more homogenous, predictable and favorable way to treatment [5].

A promising aspect of the yet incompletely understood pathophysiology of HFpEF is the presence of electrical dyssynchrony, because of existing treatments targeting this phenomenon. Electrical dyssynchrony refers to the dyssynchronous activation of the left ventricle (LV) versus the right ventricle, or within different segments of the LV. It is an important factor contributing to the progression of HF and LV remodeling in various populations [6]. While treatment of electrical dyssynchrony, also known as cardiac resynchronization therapy (CRT) or conduction system pacing, is extensively studied in HF patients with reduced ejection fraction (HFrEF) [6], [7], the potential benefit in HFpEF is gaining attention [8].

QRS duration and morphology on 12-lead electrocardiograms are traditionally used to asses electrical dyssynchrony. Prolonged QRS duration, and thus ventricular conduction delay (VCD), indicates dyssynchrony of contraction and relaxation between the left and right ventricles. Prior studies have investigated the prevalence and prognostic value of an VCD in different HFpEF populations showing a wide range from 10 % to 60 % [9], [10], [11], [12], [13], [14]. These inconsistent results are due to the usage of different cut off values of QRS duration and various HFpEF populations [9], [10], [11], [14].

Electrical dyssynchrony can also be assessed by vectorcardiography (VCG), which is a three-dimensional visualization of the electrical vectors of the heart. For dyssynchrony in particular, the vectorcardiographic measurement of interest is QRS area, which reflects LV intra-ventricular electrical dyssynchrony and identifies delayed LV lateral wall activation [15], [16], [17]. A prior study showed that QRS area is prognostically significant in patients with a QRS duration under 150 ms, suggesting that delayed LV lateral wall activation may be present and treatable with CRT, even without significant QRS widening [18]. It is speculated that QRS area increases prior to QRS duration and therefore identifies electrical dyssynchrony earlier than QRS duration. This makes QRS area interesting in HFpEF patients, as well, since the relative infrequency of wide QRS intervals in this patient group [19]. Currently, the incidence and prognostic value of an increased QRS area is unknown in HFpEF patients. If an increased QRS area is indeed related to worse prognosis in HFpEF patients, CRT might be a therapeutic option in this specific subgroup of HFpEF patients with LV intra-ventricular electrical dyssynchrony [20].

Our hypothesis is that HFpEF patients with LV electrical dyssynchrony present an isolated phenotype with a different pathophysiology and worse prognosis in comparison to HFpEF patients without LV electrical dyssynchrony, which yields therapeutic opportunities. The current study assesses the incidence and characteristics of clinical phenotypes of HFpEF patients with VCD and LV electrical dyssynchrony, assessed by QRS duration and QRS area and its association with adverse clinical outcomes.

## 2 Methods

### 2.1 Research design and study population

This single-center study is part of an ongoing prospective cohort study in the Netherlands [21]. Patient inclusion started in January 2015 at the Maastricht University Medical Center (MUMC+), a secondary and tertiary care center. Patients suspected of HFpEF or suffering from unexplained dyspnea with a left ventricular ejection fraction of more than 50 % were referred to a specialized HFpEF outpatient clinic either from within the MUMC+, nearby hospitals or general practitioners. Patients systematically underwent a routine diagnostic work-up to assess HF status, risk factors and comorbidities. The likelihood of HFpEF was estimated by three different diagnostic scores: the Heart Failure Association Pre-test assessment, Echocardiography & natriuretic peptide, Functional testing, Final aetiology (HFA-PEFF) score [22], the H_2_FPEF score [23], and the European Society of Cardiology clinical HF guideline [24]. Additional diagnostic tests, such as right-sided heart catheterization or invasive coronary angiography were performed in case of clinical uncertainty. In some cases, patients met the criteria of HFpEF but were diagnosed with a cardiomyopathy, haemodynamically significant congenital disease, restrictive cardiomyopathies, constrictive pericarditis, severe valvular heart disease or a history of heart transplantation, and thus were not diagnosed with HFpEF. The final diagnosis was based on the European society of cardiology guidelines of 2016 and discussed during weekly meetings and approved if consensus was reached among a minimum of two HF specialists.

For this sub-study only patients diagnosed with HFpEF were included. Patients with a pacemaker or implantable cardioverter defibrillator (ICD) at baseline were excluded. Written informed consent was provided by all included patients and the Medical Ethics Review Committee of MUMC + approved the study (NCT04976348). This study adhered to the Principles of the Declaration of Helsinki. At baseline, the following data were collected: demographics, medical history, clinical data, biological data, medication usage, electrocardiographic data and echocardiographic data. At one year follow-up patients were recalled to the outpatient clinic and underwent echocardiography, electrocardiography (ECG) and biomarker assessment.

### 2.2 Electrocardiography

Standard 12-lead ECG recordings were obtained at baseline. Recordings more than 6 months prior baseline or than 3 months after baseline were excluded for analysis. The collected recordings were analysed in the MUSE Cardiology Information System version 8.0.2.10132 with 12SL analysis software version 241 (GE Healthcare, Milwaukee, WI). VCD was scored as present if any of these ECG recordings showed a QRS duration ≥120 ms based on automated analysis. The remaining patients were categorised into those with a QRS duration between 100 ms and 119 ms and those with a QRS duration of less than 100 ms.

### 2.3 Vectorcardiography

**Fig. 1:**
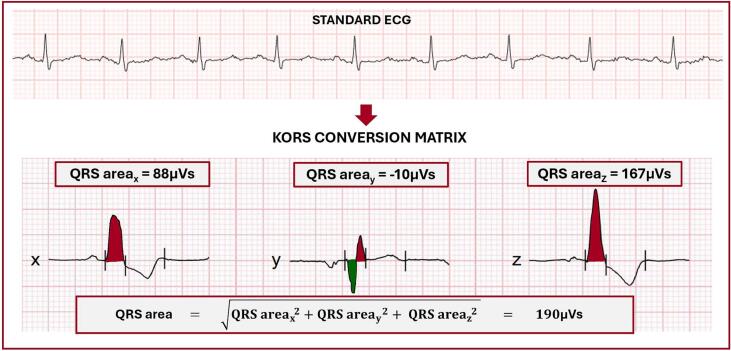
**Calculation of QRS area from the vectorcardiogram.** QRS area is calculated using the integral between the ventricular deflection curve and the baseline of the QRS complex.

Vectorcardiograms were derived from standard 12-lead ECGs using customized MATLAB software (MathWorks, Inc., Natick, MA) using the Kors method [17], [18]. A vectorcardiogram consists of three orthogonal leads (X, Y and Z), which forms a 3D vector loop. By calculating the integral between the ventricular deflection curve and the baseline of the QRS in each lead, the QRS area could be determined (Fig. 1) [17]. QRS area measurements were obtained by two investigators. Two groups were formed based on a cut-off value of 43.1µVs, which represents the third quartile of QRS area in our cohort. Binning analyses were performed to determine a suitable cut off value for QRS area without success. We conducted receiver operating characteristic analyses and tried calculating the Youden’s index to determine the optimal cut-off point for QRS area in prognostic analyses. However, the area under the curve was below 0.6, indicating that QRS area is not a reliable predictor of outcomes and that defining a specific cut-off value was not feasible. By using the third quartile as cut off value, the group distribution is more similar to those based on QRS duration cutoff of 120 ms. Because a QRS area ≥69 µVs diagnoses delayed LV lateral wall activation in HFrEF patients [17], we did not subdivide the group of patients with QRSarea ≤ 43.1. Prevalence of a QRS area ≥69 µVs was also separately evaluated.

### 2.4 Outcomes

Adverse clinical outcomes were assessed as all-cause mortality and HF hospitalization. The primary endpoint was a combination of all-cause mortality or HF hospitalization. For all non-fatal events, only the first event was included in the analyses. Clinical outcomes were manually assessed through electronic hospital records in December 2023.

### 2.5 Statistical analyses

Descriptive analyses were performed to compare baseline demographics, comorbidities, clinical and biological data, medication usage and echocardiographic and ECG measurements between the different groups based on QRS duration and QRS area. Continuous variables were expressed as mean ± standard deviation if normally distributed (tested with visual assessment of QQ-plots and Levenes test of equal variances) and differences were tested using one-way ANOVA or the students T-test, depending on number of groups compared. Continuous variables were expressed as median and quartiles [Q1-Q3] if not normally distributed, and tested using Kruskal-Wallis or Mann-Whitney U, as appropriate. Categorical variables, presented as number and percentage, were compared using the Chi^2^ test or Fisher exact test.

Since QRS area was calculated by two different investigators, intraclass correlation coefficient (ICC) estimates and their 95 % confident intervals (CI) were calculated based on multiple raters, absolute-agreement, two-way mixed-effects model for inter-rater reliability. The inter-rater was excellent (ICC of 0.999 (95 % CI 0.993-1.000).

For the prognostic analyses, Kaplan-Meier curves were used to visualize adverse outcomes (mortality and/or HF hospitalization) and were tested using log-rank statistics. Log rank pairwise comparisons were run to determine which groups had different survival distributions, with Bonferroni correction for multiple testing. If the Kaplan-Meier curves intersected, in addition to a log-rank test, a Breslow and Tarone test was performed. Furthermore, a univariable and multivariable-adjusted Cox proportional-hazards regression analyses was conducted. To account for the effect of age over time, we included a time-varying covariate interaction for age. Furthermore, given the non-linearity observed in the NTproBNP values, we used the natural logarithm of NTproBNP (log NTproBNP) in our model. To better elucidate the effect of QRS duration, we grouped QRS duration in 5 ms increments instead of using it as a continuous variable. Multivariable-adjusted models included variables that significantly contributed to the combined outcome in the univariable model and was adjusted for age and sex. Cubic spline plots were generated to visualize the relationship between the QRS duration and the hazard ratio (HR) for all-cause mortality and HF hospitalizations.

All statistical analyses were performed by means of RStudio version 2021.09.01 (Boston, MA, USA). A two-tailed p-value of less than 0.05 was considered statistically significant.

## 3 Results

### 3.1 Baseline characteristics

Between January 2015 and December 2020, we enrolled a total of 520 patients, of whom 382 received a diagnosis of HFpEF. After excluding patients with a pacemaker or ICD at baseline (n = 31) or missing baseline ECG (n = 2), 349 HFpEF patients were included in this analysis (mean age 75 years; 69 % female; Supplemental Fig. S1).

Regarding ECG findings, 243 (70 %) patients had a QRS duration < 100 ms, 74 (21 %) a QRS duration between 100–119 ms, and 32 (9 %) a QRS duration ≥ 120 ms. Significantly more male patients presented with a QRS duration of ≥ 100 ms compared to female patients (p < 0.001, Table 1). With increasing QRS duration, HFpEF patients presented with more advanced disease (Table 1). This trend was evident as patients with longer QRS durations exhibited significantly more atrial fibrillation/flutter, a higher NT-proBNP at baseline, lower LV ejection fraction (LVEF), higher LV end diastolic diameter, and higher LV mass index. Furthermore, a significant trend of increasing QRS area with longer QRS duration was observed (Table 1).

**Table 1:** Baseline characteristics of HFpEF patients by QRS duration.

**Characteristic**	**Valid,** ***n***	**QRS < 100msn = 243**	**Valid,** ***n***	**QRS 100-119msn = 74**	**Valid,** ***n***	**QRS >= 120msn = 32**	**p-value for trend**
Female sex	243	193 (79 %)	74	32 (45 %)	32	13 (41 %)	< 0.001
Age (years)	243	75 (±7)	74	75 (± 7)	32	75 (± 9)	0.840
**HFpEF scores:**							
HFA-PEFF	243	5 [4], [5], [6]	74	5 [4], [5], [6]	32	5 [5], [6]	0.175
H2FPEF	243	6 [4], [5], [6], [7]	74	6 [5], [6], [7], [8]	32	6 [5], [6], [7]	0.123
**Medical history:**							
DM2	243	58 (24 %)	74	23 (31 %)	32	8 (25 %)	0.459
Kidney disease	243	68 (28 %)	74	18 (24 %)	32	9 (28 %)	0.820
Atrial fibrillation/flutter	243	150 (62 %)	74	53 (72 %)	32	28 (88 %)	0.008
Hypertension	243	187 (77 %)	74	55 (74 %)	32	14 (44 %)	<0.001
Significant CAD	243	48 (20 %)	74	20 (27 %)	32	8 (25 %)	0.372
**Physical examination:**							
BMI (kg/m^2^)	243	30.1 (± 5.5)	74	31.4 (± 6.4)	32	30.7 (±6.0)	0.261
SBP (mmHg)	230	149 (23)	66	152 (± 23)	30	148 (±23)	0.641
DBP (mmHg)	229	78 (± 13)	66	78 (± 12)	30	76 (± 10)	0.568
**Biological data:**							
NT-proBNP (pg/ml)	243	516 [237–1387]	74	558 [313–1133]	32	871 [567–1861]	0.019
eGFR (ml/min/1.73 m^2^)	234	55.4 (± 18.8)	72	55.7 (± 19.1)	29	47.0 (±18.4)	0.069
**Echocardiography:**							
LVEF (%)	242	61 (± 6)	74	58 (± 5)	32	59 (± 7)	0.001
LVEDD (mm)	241	47 (± 5)	74	50 (±6)	32	49 (± 6)	< 0.001
LVIVSd (mm)	240	9 (± 1)	74	10 (± 1)	32	10 (± 1)	< 0.001
LVPWd (mm)	239	9 (± 1)	74	10 (± 1)	32	10 (±1)	< 0.001
RWT	239	0.39 (± 0.06)	74	0.39 (± 0.07)	32	0.40 (± 0.06)	0.499
LVMI (g/m^2^)	239	76 (± 18)	74	87 (±20)	32	92 (± 23)	< 0.001
LAVI (ml/m^2^)	234	49 (± 19)	72	49 (±14)	28	50 (± 14)	0.912
E/e’ average	175	11.5 (± 4.0)	50	11.6 (± 4.4)	20	13.5 (± 5.4)	0.116
TR velocity (m/s)	226	2.6 (± 0.4)	64	2.7 (± 0.5)	30	2.9 (± 0.6)	0.001
**Electrocardiography:**							
HR (beats/min)	243	72 (± 13)	74	71 (± 13)	32	70 (± 11)	0.388
QRS duration (ms)	243	84 [80–92]	74	104 [100–108]	32	132 [124–140]	< 0.001
**Vectorcardiography:**							
QRS area (µVs)	240	30.1 [20.9–38.7]	73	34.9 [25.0–47.9]	32	46.8 [27.2–72.3]	< 0.001
QRS area > 43.1µVs	240	44 (18 %)	73	26 (35.1 %)	32	17 (53.1 %)	< 0.001
**Medication:**							
Beta blocker	240	181 (75 %)	74	50 (68 %)	32	24 (75 %)	0.401
Amiodarone	247	4 (2 %)	70	1 (1 %)	30	3 (10.0 %)	0.046

Data are presented as n (%), mean ± SD or median [interquartile range].

**Abbreviations:** HFA-PEFF, the Heart Failure Association Pre-test assessment, Echocardiography & natriuretic peptide, Functional testing, Final aetiology score; DM2, diabetes mellitus type two; CAD, coronary artery disease, BMI, body mass index; SBP, systolic blood pressure; DBP, diastolic blood pressure; NT-proBNP, N-terminal pro b-type natriuretic peptide; GFR, glomerular filtration rate; LVEF, left ventricular ejection fraction; LVEDD, left ventricular end diastolic diameter; LVIVSd, left ventricular interventricular septum diameter; LVPWd, left ventricular posterior wall diameter; RWT, relative wall thickness; LVMI, left ventricular mass index; LAVI, left atrial volume index; TR, tricuspid regurgitation; HR, heart rate.

Regarding VCG findings, the median baseline QRS area was 32.2 µVS [21.5 µVs – 43.1 µVs]. The third quartile value of 43.1µVs was used to divide the cohort in two groups (Table 2). Within these groups, 258 patients (74 %) exhibited a QRS area of ≤43.1 µVs, while 87 patients (25 %) displayed a QRS area exceeding 43.1 µVs. Only 14 (4 %) patients had a QRS area ≥69 µVs, which diagnoses delayed LV lateral wall activation in HFrEF patients (17). The classification of patients based on QRS area revealed comparable yet less pronounced baseline distinctions compared to categorization by QRS duration (Table 2).

**Table 2:** Baseline characteristics of HFpEF patients by QRS area.

**Characteristic**	**Valid,** ***n***	**QRS area ≤ 43.1µVsn = 258**	**Valid,** ***n***	**QRS area > 43.1µVsn = 87**	**p-value**
Female sex	258	177 (69 %)	87	58 (67 %)	0.840
Age (years)	258	75 (± 7)	87	75 (± 7)	0.724
**HFpEF scores:**					
HFA-PEFF	258	5 [4], [5], [6]	87	5 [4], [5], [6]	0.695
H2FPEF	258	6 [4], [5], [6], [7]	87	6 [5], [6], [7], [8]	0.082
**Medical history:**					
DM2	258	68 (26 %)	87	19 (22 %)	0.486
Kidney disease	258	72 (28 %)	87	21 (24 %)	0.585
Atrial fibrillation/flutter	258	160 (62 %)	87	68 (78 %)	0.009
Hypertension	258	193 (75 %)	87	60 (69 %)	0.355
Significant CAD	258	48 (19 %)	87	28 (32 %)	0.013
**Physical examination:**					
BMI (kg/m^2^)	258	30.6 (± 5.7)	87	30.1 (± 5.9)	0.572
SBP (mmHg)	244	150 (± 23)	82	152 (± 22)	0.403
DBP (mmHg)	243	78 (± 12)	82	77 (± 12)	0.770
**Biological data:**					
NT-proBNP (pg/ml)	258	566 [268 – 1412]	87	463 [286–––1294]	0.746
eGFR (ml/min/1.73 m^2^)	232	54.7 (± 19.1)	85	56.0 (± 17.9)	0.494
**Echocardiography:**					
LVEF (%)	257	61 (± 6)	87	59 (± 6)	0.250
LVEDD (mm)	256	47 (± 5)	87	49 (± 6)	0.033
LVIVSd (mm)	256	9 (± 1)	87	10 (± 2)	< 0.001
LVPWd (mm)	254	9 (± 1)	87	10 (± 2)	< 0.001
RWT	254	0.39 (± 0.06)	87	0.40 (± 0.07)	0.051
LVMI (g/m^2^)	254	77 (± 18)	87	89 (± 21)	< 0.001
LAVI (ml/m^2^)	248	48 (± 16)	82	51 (± 19)	0.172
E/e’ average	188	11.6 (± 4.2)	56	11.8 (± 4.3)	0.860
TR velocity (m/s)	236	2.7 (± 0.5)	80	2.7 (± 0.5)	0.556
**Electrocardiography:**					
HR (beats/min)	258	73 (± 12)	87	67 (± 11)	< 0.001
QRS duration (ms)	258	91 (± 14)	87	103 (± 20)	< 0.001
**Medication:**					
Beta blocker	256	183 (71 %)	86	69 (79 %)	0.146
Amiodarone	256	5 (2 %)	86	3 (4 %)	0.420

Data are presented as n (%), mean ± SD or median [interquartile range].

**Abbreviations:** HFA-PEFF, the Heart Failure Association Pre-test assessment, Echocardiography & natriuretic peptide, Functional testing, Final aetiology score; DM2, diabetes mellitus type two; CAD, coronary artery disease; BMI, body mass index; SBP, systolic blood pressure; DBP, diastolic blood pressure; NT-proBNP, N-terminal pro b-type natriuretic peptide; GFR, glomerular filtration rate; LVEF, left ventricular ejection fraction; LVEDD, left ventricular end diastolic diameter; LVIVSd, left ventricular interventricular septum diameter; LVPWd, left ventricular posterior wall diameter; RWT, relative wall thickness; LVMI, left ventricular mass index; LAVI, left atrial volume index; TR, tricuspid regurgitation; HR, heart rate.

### 3.2 One year follow-up

Data regarding echocardiography and laboratory parameters at one year follow-up are shown in Supplemental Table S1. Echocardiography conducted at the outpatient clinic after one year showed no significant difference in the absolute change in LVEF between baseline and one year follow-up among QRS duration groups (absolute LVEF delta of 6 %, 6 % and 8 % respectively, p = 0.218), neither among QRS area groups (absolute LVEF delta of 6 % and 7 % respectively, p = 0.062). Nevertheless, a significant higher proportion of patients with a QRS duration ≥120 ms had a LVEF of less than 50 % at one year follow up compared to patients with baseline QRS duration <120 ms (6 % vs. 1 %; Supplemental Table S1).

### 3.3 Adverse outcomes

During a median follow up of three years, 103 (30 %) patients reached the primary composite endpoint including HF hospitalization and/or all-cause mortality. In total 52 (15 %) patients experienced a HF hospitalization and 79 (23 %) patients died.

**Fig. 2:**
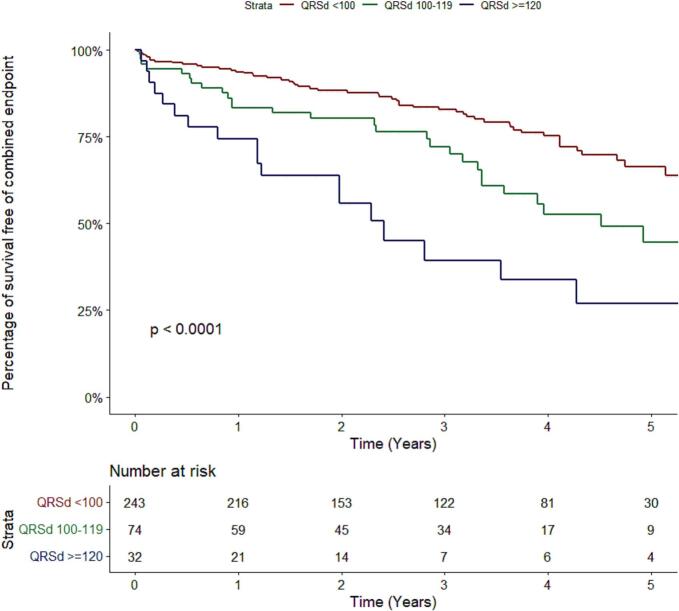
**Primary Outcome, a composite of all-cause mortality or hospitalization for heart Failure in relation to QRS duration.** Pairwise comparisons: QRS duration < 100 ms vs QRS duration 100–119 ms, p = 0.007; QRS duration 100–119 ms vs QRS duration ≥ 120 ms, p = 0.009; QRS duration < 100 ms vs QRS duration ≥ 120 ms, p < 0.001 **Abbreviations:** QRSd, QRS duration.

The primary composite endpoint was observed in 23 % of patients with a QRS duration <100 ms, 39 % with a QRS duration 100–119 ms and 59 % with a QRS duration ≥120 ms (log rank p < 0.001; Fig. 2). Pairwise comparisons were significantly different between all three QRS duration groups. When analysing the single components of the primary composite endpoint, HF hospitalization incidence was higher in patients with QRS duration ≥120 ms compared to QRS duration <100 ms (p < 0.001) and 100–119 ms (p < 0.001) (Supplemental Fig. S2), and all-cause mortality was higher in both QRS duration ≥120 ms and 100–119 ms compared to <100 ms (p = 0.027 and p = 0.010 respectively; Supplemental Fig. S3). After adjustments for age and sex, QRS duration remained a significant predictor of adverse outcome; composite endpoint HR 1.12 (95 % CI 1.03 – 1.21) per 5 ms increase, p = 0.003; Table 3). This effect was driven by HF hospitalizations (Supplemental Table S2) and not by all-cause mortality (Supplemental Table S3). Additionally, when comparing the independent effect of QRS duration on the composite outcome between males and females, a more pronounced effect was observed in males (Supplemental Tables S4 and S5).

**Table 3:** Univariable and multivariable cox regression analyses for the primary composite endpoint.

**Composite endpoint**	**Univariable analysis**			**Multivariable analysis**		
	**HR**	**95 % CI**	**p-value**	**HR**	**95 % CI**	**p-value**
Sex	1.12	0.46 – 2.74	0.800	1.19	0.49 – 2.85	0.700
Age (years)	1.04	1.01 – 1.07	0.005	1.03	1.00 – 1.06	0.069
**Medical history:**						
Hypertension	0.45	0.30 – 0.67	< 0.001	0.42	0.27 – 0.64	< 0.001
Atrial fibrillation/flutter	1.40	0.89 – 2.18	0.140	**−**	**−**	**−**
Diabetes mellitus 2	1.97	1.32 – 2.93	< 0.001	2.52	1.65 – 3.86	< 0.001
Significant CAD	1.64	1.08 – 2.49	0.020	**−**	**−**	n.s.
**Biological data:**						
Log NT-proBNP (pg/ml)	1.64	1.33 – 2.01	< 0.001	1.42	1.14 – 1.76	0.002
GFR (ml/min/1.73 m^2^)	0.98	0.97 – 0.99	0.001	**−**	**−**	n.s.
**Echocardiography:**						
LVEF (%)	0.97	0.94 – 1.00	0.052	**−**	**−**	**−**
LVMI (g/m^2^)	1.02	1.01 – 1.02	0.002	1.01	1.00–1.02	0.050
LVEDD (mm)	1.01	0.97 – 1.05	0.500	**−**	**−**	**−**
**Electrocardiography:**						
QRS duration (per 5 ms)	1.18	1.08 – 1.28	< 0.001	1.12	1.03 – 1.21	0.009
QRS area (µVs)	1.01	1.00 – 1.02	0.046	**−**	**−**	n.s.
**Interaction:**						
QRS duration (per 5 ms) * SEX	0.93	0.83 – 1.04	0.200	0.92	0.82 – 1.03	0.130

**Abbreviations:** HR, hazard ratio; CI, confidence interval; CAD, coronary artery disease; NT-proBNP, N-terminal pro b-type natriuretic peptide; GFR, glomerular filtration rate; LVEF, left ventricular ejection fraction; LVMI, left ventricular mass index; LVEDD, left ventricular enddiastolic diameter; n.s., not significant.

The univariable cubic spline plots visualized a HR of more than one for QRS durations > 95 ms in females and >105 ms in males (Fig. 4**,** Supplemental Tables S4 and S5). Cubic spline plots for HF hospitalization and all-cause mortality separately are displayed in the supplementals (Supplemental Fig. S4).

**Fig. 3:**
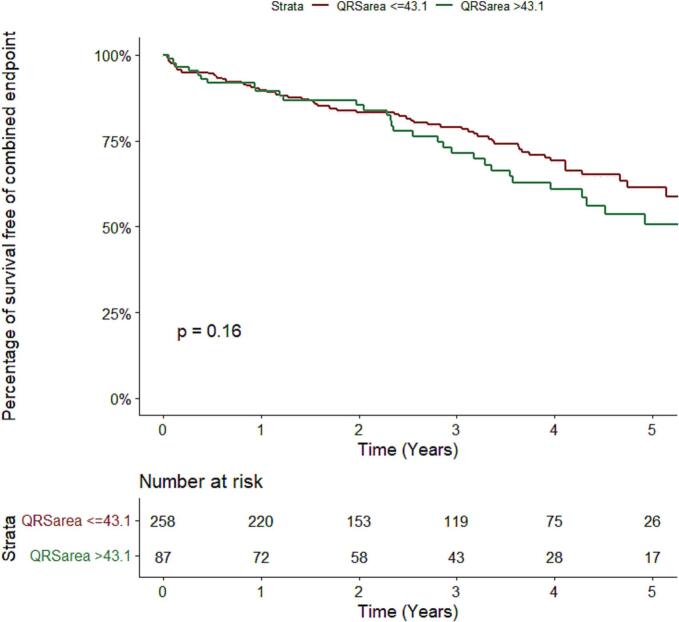
**Primary Outcome, a composite of all-cause mortality or hospitalization for heart Failure in relation to QRS area.** Tarrone-Ware test p = 0.302; Gehan-Breslow Test p = 0.448; Log rank p = 0.160.

Based on stratification by QRS area, a total of 68 (26 %) patients with QRS area ≤ 43.1 µVs and 34 (39 %) patients with QRS area >43.1 µVs experienced the primary composite endpoint. QRS area was not associated with the composite endpoint, all-cause mortality, and HF hospitalization (Fig. 3), which remained in multivariable analyses (Table 3).

**Fig. 4:**
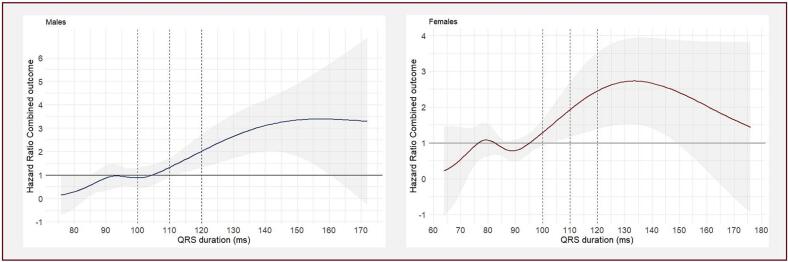
**Cubic spline plots for QRS duration in relation to the cox proportional hazards model of the combined outcome for males and females separately. Abbreviations**: ms, milliseconds.

## 4 Discussion

This study examined the incidence and prognostic value of VCD in HFpEF using QRS duration and QRS area. The prevalence of VCD in this outpatient HFpEF population was low with a QRS duration between 100–119 ms and ≥120 ms in 21 % and 9 % respectively. Despite this low prevalence, HFpEF patients with increasing QRS duration presented with more advanced disease at baseline, more often a LVEF <50 % at one year follow-up, and increased risk of adverse outcomes (HF hospitalization and all-cause mortality). The prevalence of an increased QRS area (≥69 µVs) was very low (4 %) and QRS area was not associated with adverse outcomes.

The prognostic relevance of a prolonged QRS duration in HFpEF patients found in our study, is in line with prior studies and adds several new insights. QRS duration examined by prior studies showed that a prolonged QRS duration (≥120 ms) was associated with a higher risk of cardiac death and HF hospitalization in HFpEF patients, regardless of the underlying conduction block [9], [10], [11], [12], [13], [14]. Furthermore, a QRS duration of ≥100 ms was also associated with a higher risk of mortality in hospitalized HFpEF patients [9]. These findings suggest that QRS widening could be used to identify patients with HFpEF who are at higher risk of adverse outcomes. However, these studies used previously hospitalized HF patients and different cut off values for QRS duration. Therefore, the previous findings are difficult to translate to the individual patient [9], [10], [11], [12], [13], [14]. Our study used both cut off values of 100 ms and 120 ms and our patient population consisted of ambulatory HFpEF patients from the Netherlands. The prevalence of an increased QRS duration of ≥120 ms in our study was lower than reported by other studies [10], [11], [12], [13], [14], [25], possibly related to the out-patient clinic setting which will consist of patients with less progressed HF compared to hospitalized patients. Our finding of a significant association between QRS duration ≥100 ms and clinical outcomes in ambulatory HFpEF patients is novel compared to previous studies and could have potential clinical implications. Interestingly, this study showed that patients with a higher QRS duration are more likely to develop HF with mildly reduced or reduced ejection fraction after one year. Although further research is necessary to investigate these findings, a plausible hypothesis is that HFpEF patients with a higher QRS duration may be at risk for deterioration of LVEF over time. Additionally, these patients might benefit from treatment with the “fantastic four” heart failure medications. Currently, sodium-glucose Cotransporter-2 (SGLT2) inhibitors are the only class of medications routinely prescribed for patients with HFpEF to improve outcome [26], although mineral corticosteroid antagonists are also considered in some guidelines [27]. Evidence from a pooled analysis of the DAPA-HF and DELIVER trials has demonstrated that SGLT2 inhibitors provide a significant risk reduction for adverse outcomes, regardless of the presence of VCD [28]. Similarly, spironolactone reduced adverse outcomes in the North-American sub-cohort of TOPCAT regardless of VCD presence. It remains uncertain whether other heart failure medications, such as sacubitril/valsartan [29], provide benefits specifically for HFpEF patients with VCD. Not only treatment with medication is of interest in these selected HFpEF patients with VCD, as is the use of CRT or conduction system pacing. The response to CRT appears to be dependent on the presence of systolic LV mechanical dyssynchrony (LVMD) [30]. It is proposed that delayed electrical LV lateral wall activation, which is specifically targeted by CRT, causes mechanical dysfunction [17], [30]. As such, HFpEF patients might still benefit from CRT since the relative infrequency of wider QRS intervals and the higher frequency of systolic LVMD (33 %) [31]. A QRS area of >69 µVs diagnoses delayed LV lateral wall activation with a high sensitivity and specificity in HFrEF patients [17], [32]. To date, QRS area is mainly studied to predict CRT response in HFrEF patients and predicts echocardiographic response after CRT better than current ECG criteria [33], [34], [20], [35]. The incidence and prognostic value of QRS area in HFpEF patients has not been studied yet. Our study is the first to report these findings in which the prevalence of QRS area ≥69 µVs was very low (4 %). Furthermore, we found that QRS area as a dichotomous or continuous variable was not significantly associated with adverse outcomes in HFpEF patients. QRS area is determined by a combination of specific aspects of the QRS complex, such as amplitude, duration and direction. It is supposed that presence of fibrotic tissue reduces QRS area [35], [36]. Furthermore, the lack of diastolic distensibility in HFpEF has been partially attributed to fibrosis [37], which might explain the low prevalence of increased QRS area in our study population. These findings show that the use of QRS area to identify HFpEF patients at risk of adverse outcomes is controversial. Furthermore, the use of CRT in HFpEF remains controversial and it appears to be difficult to select HFpEF patients which would benefit from CRT [38]. Nevertheless, HFpEF patients with slower average heart rate possibly benefit from higher rate pacing strategies [39], [40], and those with VCD could potentially benefit even more to improve adverse outcomes because of the higher intrinsic risk.

Although the prevalence of VCD in HFpEF is low, the pathophysiological mechanisms underlying VCD in HFpEF patients is of interest as this might lead to therapeutical options in this specific patient group. A prior study examined the relationship between QRS duration and multiple biological parameters in adult hearts and showed that prolonged QRS duration was associated with myocardial fibrosis [41]. Furthermore, it has been shown that prolonged QRS duration is associated with larger LV volumes in HF patients [42] which might be due to myocardial fibrosis. In our study, we did not find significantly more coronary artery disease in patients with higher QRS durations, suggesting that VCD was rather the result of diffuse fibrosis and not focal ischemic damage. This finding is comparable to the findings of a previous study [11]. We did, however, find significantly larger LV end diastolic diameters in patients with increasing QRS durations. Although one might argue that these differences are not clinically relevant. Moreover, we found that the prognostic value of VCD is more pronounced in males compared to females, which may be attributed to different underlying pathophysiological mechanisms and comorbidities. It is well established that male HFpEF patients more frequently have significant coronary artery disease [43], which could contribute to a higher prevalence of VCD. However, in our study, the sample sizes were too small to further investigate these differences. To determine the underlying mechanism of an increased QRS duration in HFpEF patients, further research is necessary. Furthermore, these results raise the question if VCD is a underlying pathophysiological mechanism of HFpEF or rather a consequence of more advanced disease.

### 4.1 Study limitations

This study provides insight in the prevalence and prognostic value of VCD in an ambulatory HFpEF population. We recognise certain limitations that warrant consideration when interpreting these findings. First, this study used ambulatory HFpEF patients of which some did not experience prior HF hospitalisation. Therefore, the selected population contains less advanced disease compared to a previously hospitalized HFpEF population. Nevertheless, after a median follow up of three years, 30 % reached the primary composite endpoint. Secondly, due to the low prevalence of QRS area >69 µVs in our study population, we used the cut off value of 43.1µVs to evaluate baseline differences and the prognostic value of QRS area. This cut off point had not been previously used and therefore might not have clinical significance. Nevertheless, the analyses were performed with several cut off values to create different groups (median and quartiles) and showed no difference in survival between any of these groups. Furthermore, attempts to determine a suitable cut off value of QRS area with binning analyses was not successful, indicating QRS area can’t be used to identify HFpEF patients at increased risk of adverse outcomes.

## 5 Conclusion

A prolonged QRS duration starting from 100 ms, as a marker of VCD, is an independent predictor of all-cause mortality and HF hospitalization in HFpEF. QRS area as an electrical dyssynchrony marker did not prove to be of prognostic value in HFpEF patients. Only 4 % had QRS area of ≥69 µVs, suggestive of delayed LV lateral wall activation. These findings imply that electrical dyssynchrony is not prevalent in HFpEF patients. Further studies are needed to understand the biological and mechanical mechanisms underlying the association of QRS duration and adverse outcomes, and to determine its clinical implications and role of medication and device therapies herein.

## Declaration of generative AI and AI-assisted technologies in the writing process

During the preparation of this work the author(s) used ChatGPTplus in order to improve the readability and language of the manuscript. After using this tool/service, the author(s) reviewed and edited the content as needed and take(s) full responsibility for the content of the published article.

FWP has research contracts with Medtronic, Biotronik, Abbott, Boston Scientific,

## CRediT authorship contribution statement

**Anouk Achten:** Writing – original draft, Project administration, Methodology, Investigation, Formal analysis, Data curation, Conceptualization. **Jerremy Weerts:** Writing – review & editing, Supervision, Investigation, Formal analysis, Data curation, Conceptualization. **Johan van Koll:** Writing – review & editing, Investigation. **Mohammed Ghossein:** Writing – review & editing, Software, Methodology, Data curation, Conceptualization. **Sanne G.J. Mourmans:** . **Arantxa Barandiarán Aizpurua:** . **Antonius M.W. van Stipdonk:** . **Kevin Vernooy:** Writing – review & editing, Methodology, Conceptualization. **Frits W. Prinzen:** Writing – review & editing, Visualization, Software, Methodology, Conceptualization. **Hans-Peter Brunner-La Rocca:** Writing – review & editing, Supervision. **Christian Knackstedt:** Writing – review & editing, Supervision, Funding acquisition. **Vanessa P.M. van Empel:** .

## Declaration of competing interest

The authors declare the following financial interests/personal relationships which may be considered as potential competing interests: [EBR Systems and Microport CRM. KV has research contracts and received educational grants and consultancy fees from Medtronic (Ireland), Abbot (United States); Boston Scientific (United States), Microport (China), Biosense Wespster (United States) and Phillips (The Netherlands). CK has received consultancy fees and has research contracts with Phillips (The Netherlands), TomTec Imaging Systems (Germany), Pfizer (United States) and Boehringer Ingelheim (Germany). AA has received educational grants from Pfizer (United States) and Alnylam (United States), outside the submitted work. All grants were paid to the Maastricht university institute. JW has received personal fees from Corvia Medical (United States) and Boehringer Ingelheim (Germany), outside the submitted work. Other authors have nothing to declare].
