# Supplementary material for: Prevalence and prognostic value of ventricular conduction delay in heart failure with preserved ejection fraction
Source: Int J Cardiol Heart Vasc. 2025 Jan 24;57:101622. doi: 10.1016/j.ijcha.2025.101622 (PMC11804591; doi:10.1016/j.ijcha.2025.101622)
Supplement: Supplementary Data 1 [file mmc1.docx]

Prevalence and prognostic value of ventricular conduction delay in heart failure with preserved ejection fraction

Anouk Achten MD^1^#, Jerremy Weerts MD^1^#, Johan van Koll MD^1^, Mohammed Ghossein MD^1,2^, Sanne G.J. Mourmans MD^1^, Twan A.M.W. van Stipdonk MD, PhD^1^, Kevin Vernooy MD, PhD^1,2^, Frits W. Prinzen PhD^2^, Hans-Peter Brunner-La Rocca MD^1^, Christian Knackstedt MD, PhD^1^, Vanessa P.M. van Empel MD, PhD^1^

**Supplementals**

**Supplemental tables**

**Supplemental Table 1: Echocardiography and lab parameters at one year follow-up of HFpEF patients by QRS duration at baseline.**

| **Characteristic at 1 year follow up** | **Valid, *n*** | **QRS <100ms n= 243** | **Valid, *n*** | **QRS 100-119ms n= 74** | **Valid, *n*** | **QRS >= 120ms n= 32** | **p-value for trend** |
| --- | --- | --- | --- | --- | --- | --- | --- |
| **Biological data:** |  |  |  |  |  |  |  |
| NT-proBNP (pg/ml) | 154 | 548 [254-1319] | 53 | 744 [288–1311] | 22 | 1184 [714–2588] | 0.004 |
| eGFR   (ml/min/1.73m²) | 149 | 54.6 (± 19.0) | 53 | 49.4 (± 18.5) | 21 | 44.2 (±14.6) | 0.024 |
| **Echocardiography:** |  |  |  |  |  |  |  |
| LVEF (%) | 152 | 60 (± 6) | 74 | 59 (± 6) | 21 | 55 (± 9) | 0.001 |
| LVEF <50% | 152 | 8 (3%) | 52 | 5 (7%) | 21 | 5 (16%) | 0.018 |
| LVEF <40% | 152 | 0 (0%) | 52 | 1 (1%) | 21 | 2 (6%) | 0.007 |
| LVEF [Δ] | 152 | 6 (± 4) | 52 | 6 (± 5) | 21 | 8 (± 5) | 0.218 |
| LVEDD (mm) | 152 | 47 (± 6) | 52 | 48 (± 6) | 21 | 50 (± 6) | 0.119 |
| LVMI (g/m²) | 152 | 76.7 (± 20.5) | 52 | 78.3 (± 18.8) | 21 | 88.1 (± 19.3) | 0.052 |
| LAVI (ml/m²) | 150 | 48 (± 18) | 50 | 50 (± 19) | 20 | 55 (± 23) | 0.239 |
| E/e’ average | 129 | 11.3 (± 4.1) | 43 | 11.3 (± 4.2) | 16 | 13.4 (± 3.7) | 0.150 |
| TR velocity (m/s) | 146 | 2.6 (± 0.4) | 47 | 2.7 (± 0.4) | 22 | 3.1 (± 0.4) | < 0.001 |
| **Vectorcardiography:** |  |  |  |  |  |  |  |
| QRS area (µVs) | 148 | 29.9 [19.9-41.7] | 51 | 35.5 [21.9-47.8] | 20 | 40.8 [25.5-58.2] | 0.033 |
| QRS area >43.1µVs | 148 | 31 (13%) | 51 | 16 (22%) | 20 | 10 (31%) | 0.013 |

Data are presented as n (%), mean ± SD or median [interquartile range].

**Abbreviations**: NT-proBNP, N-terminal pro b-type natriuretic peptide; GFR, glomerular filtration rate; LVEF, left ventricular ejection fraction; [Δ], absolute delta value; LVEDD, left ventricular end diastolic diameter; LVIVSd, left ventricular interventricular septum diameter; LVPWd, left ventricular posterior wall diameter; LVMI, left ventricular mass index; LAVI, left atrial volume index; TR, tricuspid regurgitation.

**Supplementals Table 2: Univariable and multivariable cox regression analyses for heart failure hospitalization.**

| **Heart failure hospitalization** | **Univariable analysis** | | | **Multivariable analysis** | | |
| --- | --- | --- | --- | --- | --- | --- |
|  | **HR** | **95% CI** | **p-value** | **HR** | **95% CI** | **p-value** |
| Sex | 0.49 | 0.28 – 0.85 | 0.011 | 0.61 | 0.35 – 1.08 | 0.093 |
| Age (years) | 1.03 | 0.99 – 1.07 | 0.200 | 1.01 | 0.96 – 1.06 | 0.700 |
| **Medical history:** |  |  |  |  |  |  |
| Hypertension | 0.42 | 0.24 – 0.73 | 0.002 | 0.41 | 0.23 – 0.76 | 0.004 |
| Atrial   fibrillation/flutter | 2.38 | 1.16 – 4.89 | 0.018 | **-** | **-** | n.s. |
| Diabetes mellitus 2 | 2.56 | 1.48 – 4.42 | <0.001 | 3.20 | 1.80 – 5.68 | <0.001 |
| Significant CAD | 1.95 1.10 – 3.45 | 1.10 – 3.45 | 0.022 | **-** | **-** | n.s. |
| **Biological data:** |  |  |  |  |  |  |
| Log NT-proBNP (pg/ml) | 1.94 | 1.45 – 2.60 | <0.001 | 1.67 | 1.22 – 2.29 | 0.001 |
| GFR (ml/min/1.73m²) | 0.98 | 0.96 – 0.99 | 0.005 | **-** | **-** | n.s. |
| **Echocardiography:** |  |  |  |  |  |  |
| LVEF (%) | 0.95 | 0.91 – 1.00 | 0.037 | **-** | **-** | n.s. |
| LVMI (g/m²) | 1.02 | 1.01 – 1.03 | 0.006 | **-** | **-** | n.s. |
| LVEDD (mm) | 1.04 | 0.98 – 1.09 | 0.2 | **-** | **-** | **-** |
| **Electrocardiography:** |  |  |  |  |  |  |
| QRS duration (per 5ms) | 1.03 | 1.01 – 1.04 | <0.001 | 1.09 | 1.02 – 1.18 | 0.013 |
| QRS area (µVs) | 1.00 | 0.99 – 1.02 | 0.700 | **-** | **-** | - |

**Abbreviations**: HR, hazard ratio; CI, confidence interval; CAD, coronary artery disease; NT-proBNP, N-terminal pro b-type natriuretic peptide; GFR, glomerular filtration rate; LVEF, left ventricular ejection fraction; LVMI, left ventricular mass index; LVEDD, left ventricular enddiastolic diameter; n.s., not significant

**Supplementals Table 3: Univariable and multivariable cox regression analyses for all-cause mortality.**

| **All-cause mortality** | **Univariable analysis** | | | **Multivariable analysis** | | |
| --- | --- | --- | --- | --- | --- | --- |
|  | **HR** | **95% CI** | **p-value** | **HR** | **95% CI** | **p-value** |
| Sex (female) | 0.54 | 0.34 – 0.84 | 0.006 | 0.52 | 0.33 – 0.82 | 0.005 |
| Age (years) | 1.03 | 1.01 – 1.06 | 0.015 | 1.03 | 1.00 – 1.06 | 0.022 |
| **Medical history:** |  |  |  |  |  |  |
| Hypertension | 0.47 | 0.30 – 0.75 | 0.001 | 0.43 | 0.27 – 0.69 | 0.001 |
| Atrial   fibrillation/flutter | 1.11 | 0.68 – 1.82 | 0.700 | **-** | **-** | - |
| Diabetes mellitus 2 | 1.68 | 1.06 – 2.65 | 0.027 | 2.04 | 1.27 – 3.28 | 0.003 |
| Significant CAD | 1.58 | 0.98 – 2.54 | 0.059 | **-** | **-** | - |
| **Biological data:** |  |  |  |  |  |  |
| Log NT-proBNP (pg/ml) | 1.51 | 1.20 – 1.90 | <0.001 | 1.34 | 1.05 – 1.71 | 0.017 |
| GFR (ml/min/1.73m²) | 0.99 | 0.97 – 1.00 | 0.031 | **-** | **-** | n.s. |
| **Echocardiography:** |  |  |  |  |  |  |
| LVEF (%) | 0.99 | 0.95 – 1.02 | 0.400 | **-** | **-** | - |
| LVMI (g/m²) | 1.01 | 1.00 – 1.02 | 0.011 | **-** | **-** | n.s. |
| LVEDD (mm) | 1.00 | 0.96 – 1.04 | 1.000 | **-** | **-** | **-** |
| **Electrocardiography:** |  |  |  |  |  |  |
| QRS duration (per 5ms) | 1.13 | 1.02 – 1.25 | 0.017 | **-** | **-** | n.s. |
| QRS area (µVs) | 1.01 | 1.00 – 1.02 | 0.13 | **-** | **-** | - |

**Abbreviations**: HR, hazard ratio; CI, confidence interval; CAD, coronary artery disease; NT-proBNP, N-terminal pro b-type natriuretic peptide; GFR, glomerular filtration rate; LVEF, left ventricular ejection fraction; LVMI, left ventricular mass index; LVEDD, left ventricular enddiastolic diameter; n.s., not significant

**Supplementals Table 4: Univariable and multivariable cox regression analyses for the combined endpoint of heart failure hospitalization and all-cause mortality in males.**

| **Composite endpoint IN MALES**  **in** | **Univariable analysis** | | | **Multivariable analysis** | | |
| --- | --- | --- | --- | --- | --- | --- |
|  | **HR** | **95% CI** | **p-value** | **HR** | **95% CI** | **p-value** |
| Age (years) | 1.02 | 0.98 – 1.06 | 0.300 | 1.01 | 0.97 – 1.06 | 0.600 |
| **Medical history:** |  |  |  |  |  |  |
| Hypertension | 0.57 | 0.31 – 1.05 | 0.073 | **0.42** | **0.21** – 0.86 | **0.018** |
| Atrial   fibrillation/flutter | 0.80 | 0.40 – 1.59 | 0.500 | **-** | **-** | **-** |
| Diabetes mellitus 2 | 1.84 | 1.02 – 3.32 | 0.042 | 2.83 | 1.42 – 5.61 | 0.003 |
| Significant CAD | 1.12 | 0.60 – 2.08 | 0.700 | **-** | **-** | **-** |
| **Biological data:** |  |  |  |  |  |  |
| Log NT-proBNP (pg/ml) | 1.28 | 0.94 – 1.74 | 0.120 | **1.33** | **0.95** – 1.87 | **0.094** |
| GFR (ml/min/1.73m²) | 0.99 | 0.97 – 1.00 | 0.120 | **-** | **-** | **-** |
| **Echocardiography:** |  |  |  |  |  |  |
| LVEF (%) | 1.03 | 0.97 – 1.08 | 0.300 | **-** | **-** | **-** |
| LVMI (g/m²) | 1.03 | 1.01 – 1.05 | < 0.001 | 1.02 | 1.01 – 1.04 | 0.005 |
| LVEDD (mm) | 1.07 | 1.01 – 1.14 | 0.029 | **-** | **-** | **-** |
| **Electrocardiography:** |  |  |  |  |  |  |
| QRS duration (per 5ms) | 1.18 | 1.08 – 1.28 | < 0.001 | 1.09 | 1.0 – 1.20 | 0.058 |
| QRS area (µVs) | 1.02 | 1.01 – 1.04 | 0.005 | **-** | **-** | **-** |

**Abbreviations**: HR, hazard ratio; CI, confidence interval; CAD, coronary artery disease; NT-proBNP, N-terminal pro b-type natriuretic peptide; GFR, glomerular filtration rate; LVEF, left ventricular ejection fraction; LVMI, left ventricular mass index; LVEDD, left ventricular enddiastolic diameter; n.s., not significant

**Supplementals Table 5: Univariable and multivariable cox regression analyses for the combined endpoint of heart failure hospitalization and all-cause mortality in females.**

| **Composite endpoint IN FEMALES**  **in** | **Univariable analysis** | | | **Multivariable analysis** | | |
| --- | --- | --- | --- | --- | --- | --- |
|  | **HR** | **95% CI** | **p-value** | **HR** | **95% CI** | **p-value** |
| Age (years) | 1.05 | 1.01 – 1.09 | 0.006 | 1.03 | 0.99 – 1.07 | 0.200 |
| **Medical history:** |  |  |  |  |  |  |
| Hypertension | 0.38 | 0.22 – 0.65 | < 0.001 | 0.43 | 0.24 – 0.75 | 0.003 |
| Atrial   fibrillation/flutter | 1.69 | 0.94 – 3.05 | 0.082 | **-** | **-** | **-** |
| Diabetes mellitus 2 | 1.86 | 1.08 – 3.21 | 0.026 | 2.11 | 1.20 – 3.71  33 | 0.009 |
| Significant CAD | 1.92 | 1.09 – 3.38 | 0.025 | **-** | **-** | **-** |
| **Biological data:** |  |  |  |  |  |  |
| Log NT-proBNP (pg/ml) | 1.92 | 1.46 – 2.53 | < 0.001 | 1.67 | 1.23 – 2.22 | < 0.001 |
| GFR (ml/min/1.73m²) | 0.98 | 0.96 – 0.99 | 0.002 | **-** | **-** | **-** |
| **Echocardiography:** |  |  |  |  |  |  |
| LVEF (%) | 0.95 | 0.91 – 0.99 | 0.021 | **-** | **-** | **-** |
| LVMI (g/m²) | 1.00 | 0.99 – 1.02 | 0.600 | 1.00 | 0.99 – 1.02 | 0.900 |
| LVEDD (mm) | 0.94 | 0.89 – 1.00 | 0.038 | **-** | **-** | **-** |
| **Electrocardiography:** |  |  |  |  |  |  |
| QRS duration (per 5ms) | 1.09 | 1.01 – 1.18 | 0.029 | 1.01 | 0.94 – 1.09 | 0.800 |
| QRS area (µVs) | 1.00 | 0.99 – 1.02 | 0.800 | **-** | **-** | - |

**Abbreviations**: HR, hazard ratio; CI, confidence interval; CAD, coronary artery disease; NT-proBNP, N-terminal pro b-type natriuretic peptide; GFR, glomerular filtration rate; LVEF, left ventricular ejection fraction; LVMI, left ventricular mass index; LVEDD, left ventricular enddiastolic diameter; n.s., not significant

**Supplemental figures**


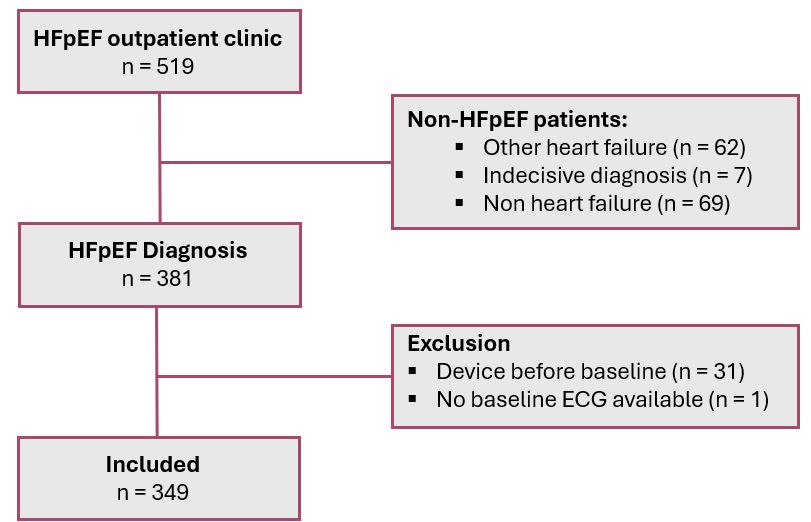


**Supplemental Figure 1**: **Flow diagram of inclusion and exclusion criteria**

**Abbreviations:** HFpEF, heart failure with preserved ejection fraction


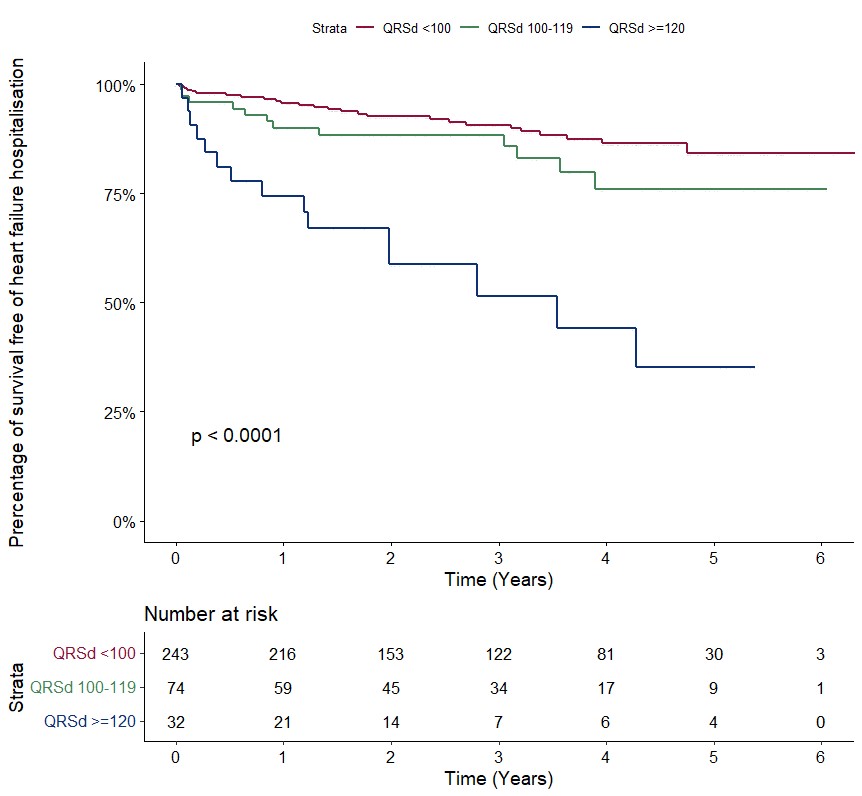


**Supplemental Figure 2**: **Hospitalization for heart Failure in relation to QRS duration.**

Pairwise comparisons:

QRS duration <100ms vs QRS duration 100-119ms, p=0.116;

QRS duration 100-119ms vs QRS duration ≥120ms, p<0.001;

QRS duration <100ms vs QRS duration ≥120ms, p<0.001

**Abbreviations:** QRSd, QRS duration


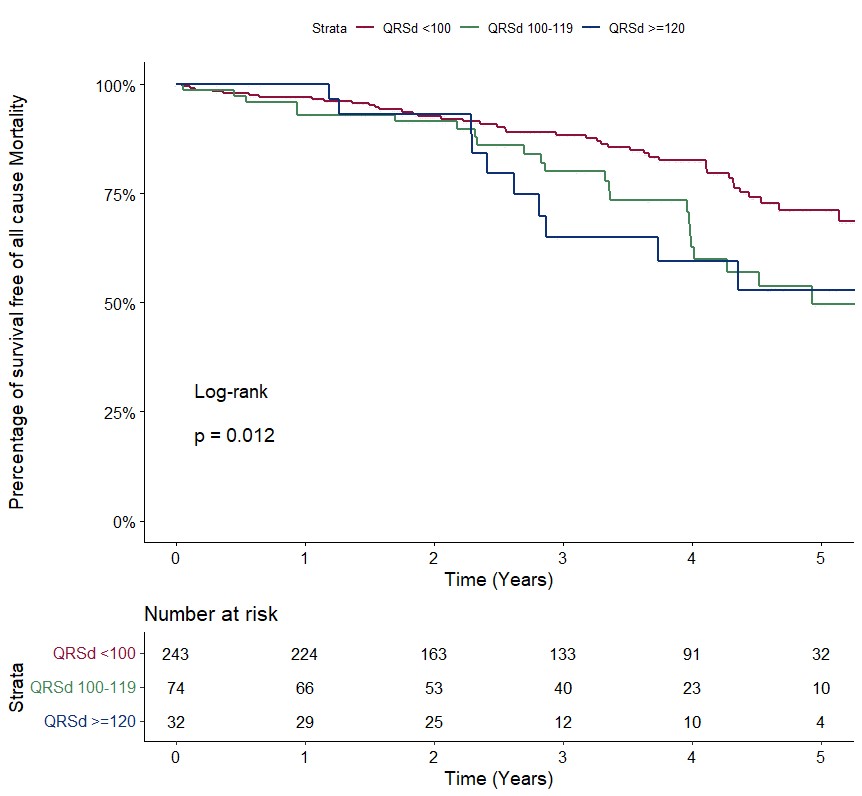


**Supplemental Figure 3**: **All-cause mortality in relation to QRS duration.**

Tarrone-Ware test p=0.027; Gehan-Breslow Test p=0.056; Log rank p=0.012

Pairwise comparisons:

QRS duration <100ms vs QRS duration 100-119ms, p=0.027;

QRS duration 100-119ms vs QRS duration ≥120ms, p=0.570;

QRS duration <100ms vs QRS duration ≥120ms, p=0.027

**Abbreviations:** QRSd, QRS duration


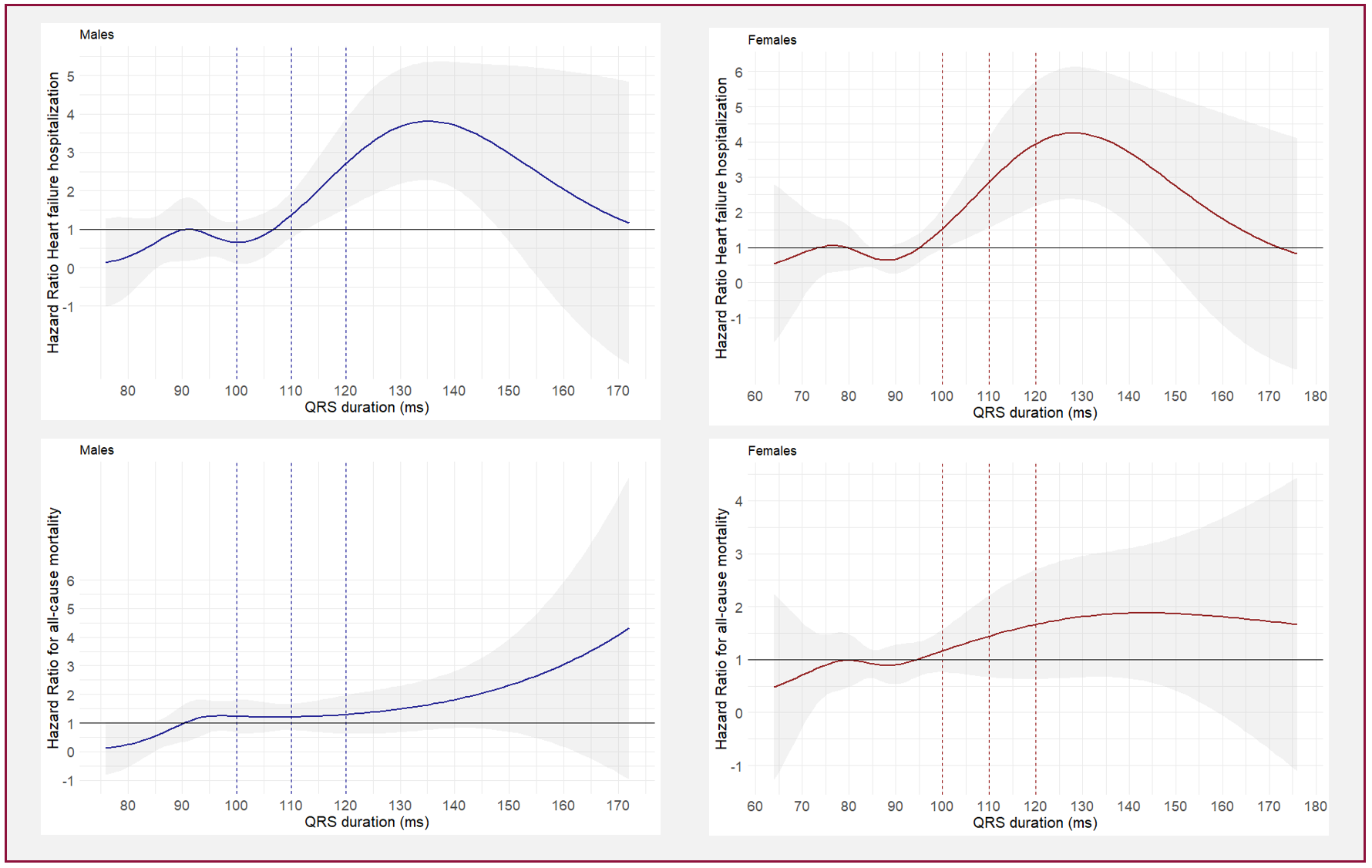


**Supplemental Figure 4: Cubic spline plots for QRS duration in relation to the cox proportional hazards model of the heart failure hospitalizations and all-cause mortality for males and females separately.**

**Abbreviations:** ms, milliseconds
